# Multidimensional Frailty and Traumatic Brain Injury among Older Adults: A Literature Review* **

**DOI:** 10.17533/udea.iee.v41n2e02

**Published:** 2023-08-18

**Authors:** Kathryn Gerber, Evelyn Iriarte, Carmen Josefa Sierra

**Affiliations:** 1 RN. Ph.D. School of Nursing and Health Studies, University of Miami, Coral Gables, Florida, U.S. Email: kgerber20@miami.edu https://orcid.org/0000-0003-0581-9536 University of Miami School of Nursing and Health Studies University of Miami Coral Gables Florida USA kgerber20@miami.edu; 2 Ph.D, MSN, RN. Adjunct Instructor at the School of Nursing, Pontificia Universidad Catolica de Chile, Santiago, Chile. Postdoctoral Fellow at the University of Colorado College of Nursing, Aurora, Colorado, U.S., and Young Researcher at Millennium Institute for Care Research, MICARE, Santiago, Chile. Email: evelyn.iriarteparra@cuanschutz.edu. https://orcid.org/0000-0002-9618-7898 Pontificia Universidad Católica de Chile School of Nursing Pontificia Universidad Catolica de Chile Santiago Chile evelyn.iriarteparra@cuanschutz.edu; 3 DNP, RN, CCTN. School of Nursing and Health Studies, University of Miami, Coral Gables, Florida, U.S. Email: carmenjsierra@miami.edu https://orcid.org/0000-0003-0581-9536 University of Miami School of Nursing and Health Studies University of Miami Coral Gables Florida USA carmenjsierra@miami.edu

**Keywords:** frail elderly, brain injuries, multiple trauma, accidental falls, Anciano frágil, lesiones encefálicas, traumatismo múltiple, accidentes por caídas, idoso fragilizado, lesões encefálicas, traumatismo múltiplo, acidentes por quedas

## Abstract

**Background:**

Numerous health conditions in the older adult population can be attributed to falls, including traumatic brain injury (TBI), which can lead to devastating short and long-term sequelae. Older adults are also more likely to experience frailty, which encompasses physical, psychological, and social deficits that may lead to adverse health outcomes. Our literature review synthesizes current evidence for understanding frailty in the context of TBI among older adults using the Integral Model of Frailty as a framework.

**Content synthesis:**

A total of 32 articles were identified, and 9 articles were included. The results of this review indicate that outcomes resulting from TBI are closely linked to the physical, psychological, and social domains of frailty.

**Conclusions:**

A small amount of literature currently examines frailty in the context of TBI among older adults. Using the Integral Model of Frailty to understand frailty in the context of TBI can help clinicians anticipate patient outcomes and improve care plans. We emphasize the need for a greater understanding of TBI concerning frailty to improve health outcomes among older adult patients.

## Resumen

**Antecedentes:**

Numerosos trastornos de salud en la población de adultos mayores pueden atribuirse a las caídas, incluida la lesión cerebral traumática (LCT), que puede provocar secuelas devastadoras a corto y largo plazo. Los adultos mayores también son más propensos a experimentar fragilidad, que abarca déficits físicos, psicológicos y sociales que pueden conducir a resultados adversos para la salud. Nuestra revisión de la literatura sintetiza la evidencia actual para la comprensión de la fragilidad en el contexto de la LCT entre los adultos mayores utilizando el Modelo Integral de Fragilidad como marco.

**Síntesis del contenido:**

Un total de 32 artículos fueron identificados, y 9 artículos fueron incluidos. Los hallazgos de esta revisión indican que los resultados de la LCT están estrechamente relacionados con los dominios físico, psicológico y social de la fragilidad.

**Conclusión:**

Una pequeña cantidad de literatura examina actualmente la fragilidad en el contexto de la LCT entre los adultos mayores. Usar el Modelo Integral de Fragilidad para entender la fragilidad en el contexto de la LCT puede ayudar a los clínicos a anticipar los resultados de los pacientes y mejorar los planes de cuidados. Enfatizamos la necesidad de una mayor comprensión de la LCT en relación con la fragilidad para mejorar los resultados de salud entre los pacientes adultos mayores.

## Resumo

**Antecedentes:**

Numerosos distúrbios de saúde na população idosa podem ser atribuídos a quedas, incluindo traumatismo cranioencefálico (TCE), que pode causar sequelas devastadoras a curto e longo prazo. Os idosos também são mais propensos a experimentar fragilidade, que engloba déficits físicos, psicológicos e sociais que podem levar a resultados adversos à saúde. Nossa revisão da literatura sintetiza as evidências atuais para entender a fragilidade no contexto do TCE entre idosos usando o Modelo Abrangente de Fragilidade como estrutura.

**Síntese de conteúdo:**

Um total de 32 artigos foram identificados e 9 artigos foram incluídos. As descobertas desta revisão indicam que os resultados do TCE estão intimamente relacionados aos domínios físico, psicológico e social da fragilidade.

**Conclusão:**

Um pequeno corpo de literatura atualmente examina a fragilidade no contexto do TCE entre adultos mais velhos. Usar o Modelo Abrangente de Fragilidade para entender a fragilidade no contexto do TCE pode ajudar os médicos a antecipar os resultados do paciente e melhorar os planos de tratamento. Enfatizamos a necessidade de uma maior compreensão do TCE em relação à fragilidade para melhorar os resultados de saúde entre pacientes idosos.

## Introduction

Globally, 727 million people were aged 65 or older in the year 2020, and in the next three decades this population is expected to more than double, reaching 1.5 billion by 2050.^1^ As older populations increase, geriatric syndromes are becoming an area of particular concern among clinicians and researchers caring for older adults. Care of older adults is complex and often involves multiple overlapping, interacting factors, including aging physiology, comorbidities, polypharmacy, and geriatric syndromes that impact on physical, social, and cognitive functioning.^2^ Geriatric syndromes such as frailty, sarcopenia, dementia, and sensory impairment are highly prevalent in older adults across all care continuums.^2^ Among them, frailty represents a unique condition of increased risk for adverse health outcomes.^2^^,^^3^ From a physical and functional approach, frailty is described as a syndrome of decreased physiological reserve and increased vulnerability to stressors due to reduced homeostatic reserves, which increases susceptibility to falls, hospital admissions, disability, and mortality.^4^^-^^6^

The most used definitions of frailty among older adults are based on the Fried Frailty Phenotype (FFP), which defines frailty as having equal to or greater than three features, including exhaustion, reduced muscle strength, low physical activity, slow walking speed, and unintentional weight loss.^5^ An alternative approach is the Cumulative Deficit Model of Frailty (CDMF), which describes frailty as a state of vulnerability rather than a syndrome and suggests that frailty arises from the cumulative effects of age-related deficits.^7^^,^^8^ The CDCM has a quantitative approach: the higher number of deficits, the more likely they contribute to an adverse health outcome.^8^

Frailty increases older adults’ susceptibility to sustain falls, leading to injuries such as fractures or traumatic brain injury (TBI).^9^ TBI is a significant concern for older adults, accounting for 80,000 Emergency Department visits per year in the geriatric population, of which 75% of patients are hospitalized. Of those older adults with TBI, 51% are due to falls.^10^ TBI may result in functional decline, cognitive deficits, and affective symptoms such as depression or anxiety.^11^^,^^12^ A TBI occurs when an outside force causes the brain to move within the skull. In older adults, the management of CPP can be challenging due to the decreased ability of the cardiovascular system to respond to shock in older adults.^13^^,^^14^ Additionally, older adults are more susceptible to sustaining TBI due to the physiological process of aging, which involves adhering the dura mater to the skull. Older adults are also increasingly likely to be receiving anticoagulant therapy, which increases susceptibility to bleeding and may increase injury severity.^10^

Older adults are susceptible to impaired healing and difficulty recovering from TBI due to immunocompromise and lower brain plasticity.^15^ Mortality has been shown to increase in TBI patients over the age of 60.^16^ TBI can also have devastating long-term consequences among older adults, including brain atrophy, demyelination, decreased blood-brain barrier function, and impaired neurogenesis, resulting in physical, cognitive, and affective symptoms.^17^^-^^18^ Geriatric patients are more likely to experience the combined impact of both TBI and frailty. Additionally, TBI has consistently been linked with future neurodegenerative diseases such as Alzheimer's Disease, which increases susceptibility to the health impacts of frailty.^19^

To better understand the complexity of frailty in the context of TBI among older adults, this study uses the Integral Model of Frailty.^20^ This model describes the multidomain frailty pathway in which frailty is considered a dynamic process whose development is influenced by several factors. The model defines frailty, such as a dynamic state affecting a person who lives losses in one or more human functioning domains (e.g., physical, psychological, and social), resulting from several factors (life-course determinants in the model), which increases the risk of adverse outcomes.^20^^-^^22^ When applied to TBI, the Integral Model of Frailty can help understand how TBI is relevant regarding frailty in physical, social, and psychological domains. 

Despite the compelling and significant need to explore frailty in the context of older adults suffering TBI, there is limited literature that uses a multidimensional approach to understand frailty. Therefore, the purpose of this literature review is to synthesize current evidence of multidimensional frailty in the context of TBI among older adults using the Integral Model of Frailty as a framework. Determining life-course determinants, domains, and adverse outcomes related to frailty may help guide future efforts and interventions to help decrease the impact of multidimensional frailty in aging populations who have suffered TBI. 

## Methods

Search Strategy, Eligibility Criteria and Information Sources. A literature review was conducted using the keywords or MESH terms [Brain Injury, Traumatic OR Traumatic Brain Injury OR Brain Trauma OR TBI (Traumatic Brain Injury) OR TBIs (Traumatic Brain Injuries)] AND [Frailty OR frail] AND [Older OR elderly OR aged]. Electronic databases searched included PubMed and Cumulative Index for Nursing and Allied Health Literature (CINAHL). Articles published in English within the past ten years (2011-2021) were selected for this literature review in order to include the most updated information. Commentaries, editorials, and dissertations were excluded. The bibliographic information resources of the University of Miami were used to extract the research articles according to the selection protocol. The methods of this literature review were guided by the Preferred Reporting Items for Systematic Reviews and Meta-Analyses (PRISMA) guidelines.^23^

**Figure 1:**
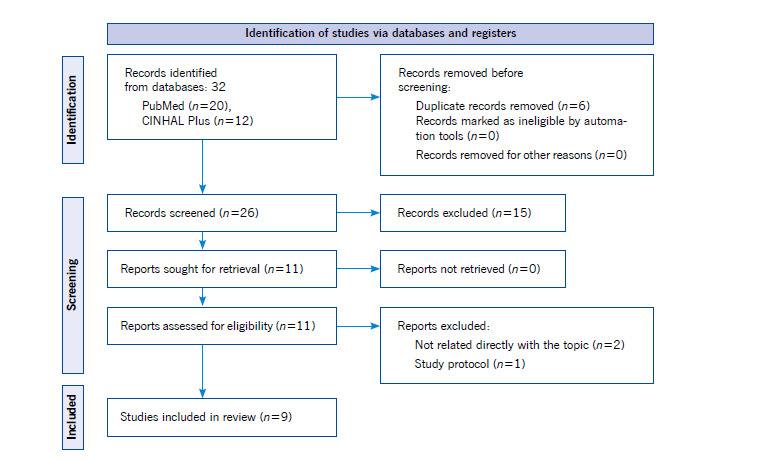
PRISMA Flowchart for the Literature Review

Selection and Data Collection Process. The information that emerged from each step of the database search was organized in Microsoft Excel (version 365) and RefWorks. Two independent reviewers (K.G. and E.I.) conducted the selection and data collection process. The first author (K.G.) carried out the initial selection and extraction process. Then, the final study sample selection was carried out by two authors independently (K.G. and E.I.). No arbitrator was required because no consensus differences arose during these processes. A flowchart of the review process based on the PRISMA-ScR is included in Figure 1. Both reviewers (K.G. and E.I.) extracted all factors from the papers that align with the literature review objective. A draft charting table in Excel was developed to record the key information of the sources. Afterward, the following data were extracted for each study included in this review: author, year, study design, setting, sample size, design/variables, and relevant results. Finally, the results identified in the selected studies were grouped according to The Integral Model of Frailty^20^ in: a) Life-Course Determinants of Frailty, b) Domains of frailty (physical, psychological, and social), and c) Adverse health outcomes related to frailty. 

Study Risk of Bias Assessment. To evaluate the risk of bias, studies were evaluated for level of evidence (e.g., RCT, quasi-experimental, non-experimental study) using the Johns Hopkins Nursing Evidence-Based Practice Rating Scale.^24^ Studies were also appraised for quality, which encompasses components of a given study that strengthen or weaken the study (e.g., study design, and retention of participants), as well as evaluating components of the study such as research question, quality of the articles included, and outcome measurements, each article being given a strong, moderate, or weak overall ranking based upon total quality appraisal score.^24^

## Results

The database results were reported using a PRISMA flowchart (see Figure 1). The results yielded from the search criteria included 12 articles from CINAHL and 20 articles from PubMed for a total of 32 studies. After removing duplicates, there were a total of 26 remaining studies. Of these studies, 18 were removed. The final remaining number of analyzed studies was 9 (Table 1). We found that the majority of studies were ranked as a 2 in the level of evidence (on a 1-5 scale, with 1 being the strongest), with either a high or good quality of research. Regarding the quality assessment rating, most studies were evaluated as strong/moderate (*n*=8; 88.9%) (Table 2).

**Table 1:** Table of Evidence of the Studies included in the Literature Review (*n*=9)

Authors	Study Site	Study Population	Study Design	Key Variables Examined / Methods	Key Findings	Level of Evidence*
Abdulle *et al*., 2018^25^	Three Dutch hospitals (Level 1 trauma centers)	Adults >60 years with mild TBI; *n*=161; mean age 70.8 (SD= 6.3)	Prospective observational cohort	Depression, anxiety, GCS, frailty	Emotional distress: frail 50%, non-frail 20%. Frailty showed to predict long-term outcomes (OR = 2.1 [95% CI: 1.59-2.77]).	IIA
Boye *et al.,* 2014^26^	Data extracted from IMPROveFALL study, conducted in the Netherlands	Adults >65 visiting emergency room due to a fall; *n*=5880; mean age 80 (SD= 8)	Descriptive, cross-sectional	Circumstances surrounding fall (e.g., location, season), injuries (e.g., TBI, open head wound, fractures)	Individuals who visited the emergency room due to a fall (n=254; 4%) sustained TBI subsequent to experiencing the fall. Falls had similar indoor (*n=*92, 52%) and outdoor (*n*=84, 48%) prevalence.	IIB
Brown *et al.,* 2017^27^	Three databases for article extraction	Adults >65; *n*=367 studies identified with 13 eligible for inclusion	Systematic review	Functioning (frailty) and health-related quality of life	Older adults had a significant trend toward lower motor FIM scores; cognitive FIM correlated with age. Studies report worse FIM for >65 than 18-64 mTBI patients. Poor health years prior to the injury and depression/fatigue shortly after injury are associated with poorer outcomes.	IIIB
DeLuca *et al.,* 2020^28^	Italy; study a part of a larger multi-centric randomized clinical trial examining cognitive tele-rehabilitation	TBI subjects: *n*=10; mean age 45.7 (SD= 14.4) and caregivers: *n*=10; mean age 43.7 (SD= 13.5)	Preliminary feasibility and usability study	Use of Telerehabilitation System VRRS (Virtual Reality Rehabilitation System), which allows for monitoring of TBI remotely at home by professional	Younger TBI patients had higher usability scores than older TBI patients. Caregivers chose more often than TBI patients to carry out the activity and had higher pressure/tension.	IIB
Harvey *et al.,* 2017^29^	New South Wales (Australia)	Fall-related TBI adults >65; *n*=6635 (20.8% (*n*=1383) were for RACF)	Retrospective study of hospital records involving data linkage between 2 time points (2008-2009 to 2012-2013)	Comparison of CD individuals with RACF residents	A higher proportion of falls for RACF when compared to CD were furniture-related (21.4% vs. 9.9%), resulted in hemorrhage (82.5% vs. 73.7%), and death (23.1% vs. 14.9%). 7.7% of hospitalizations for CD resulted in RACF placement.	IIA
Prabhakaran *et al.,* 2020^30^	Retrospective review from American College of Surgeons-Trauma Quality Improvement Project (ACS-TQIP) databank from 2014-2016.	Adults >65 who had sustained venous thromboembolism; *n*=354,272 complete records examined, VTE: *n=*4290 (1.1% of patient records); mean age 75.49 (SD= 7.02)	Retrospective review	ICU length of stay, age category, spine injury, use of a ventilator, transfusion of plasma products, severe TBI, frailty	Both frailty (*p*<0.001) and severe TBI (*p*<0.001) were independent predictors of VTE development in the elderly.	IIA
Stein *et al.,* 2011^31^	NR	NR	Literature review	Progesterone and Vitamin D hormone treatment for TBI in elderly patients	Age-related changes influence immune function and induce central nervous system changes/inflammatory response. Frailty in older adults is associated with Vitamin D deficiency. Progesterone has been shown to modulate aquaporins/reduce edema and reduce glutamate toxicity, helping to reduce cell damage/decreased function. Progesterone improves outcomes in TBI patients.	IIIB
Teo *et al.,* 2018^32^	National University Hospital of Singapore, 1200-bed acute-care, tertiary hospital	TBI adults admitted for fall >65; *n*=339; mean age 79.7 (SD= 8.0)	Retrospective medical chart review	Subdural hemorrhage, hospitalization prior to TBI, formal dx cognitive impairment or dementia, fall risk index, polypharmacy, fall circumstances (e.g., location, nature of fall), ADLs, outpatient clinic appointments	Fall-related TBI is associated with a decline in ADLs, polypharmacy, and specialist outpatient clinic appointments (*p*<0.001). Mild cognitive impairment or dementia are associated with fall-related TBI admission (3.31 [95% CI 1.68-6.51], *p*<0.001.	IIA
Tracy *et al.,* 2020^33^	American College of Surgeons Trauma Quality Improvement Program (TQIP) registry between fall 2016-spring 2019	Adults who sustained TBI >16 years of age; *n*=2352 (27.8% of patients in overall database)	Retrospective review of TQIP registry	Frailty index: patients stratified into no frailty (0), mild frailty (0.1), and moderate to severe frailty (>0.2).	Higher frailty scores were associated with increasing age (*p*<0.0001), increased rates of SNF/LTAC discharge (*p*= 0.0002) and decreasing injury severity score (*p*=0.001). Moderate-severe frailty increased acute kidney injury (OR = 2.06, [95% CI = 1.07-3.99], *p*=0.03) and any unplanned event (OR 1.6, [95% CI 1.1-2.3], *p*=0.01).	IIB

*Note.* ADL= activities of daily living; CD= community-dwelling; FIM = modified frailty index; GCS= Glasgow coma scale; ICU= intensive care unit; NR= not reported; RACF= residential aged care facility; TBI= traumatic brain injury; VTE= venous thromboembolism.

**Table 2:** Quality Assessment Appraisal

	Domains for reviews*:														
Study	1	2	3	4	5	6	7	8	9	10					Overall Score
Brown *et al*., 2017^27^	Y	Y	Y	Y	N	Y	Y	Y	N	Y					8
Stein *et al.*, 2011^31^	Y	Y	N	N	N	N	Y	N	N	Y					4
	Domains for studies**:														
	1	2	3	4	5	6	7	8	9	10	11	12	13	14	Overall Score
Abdulle *et al.*, 2018^25^	Y	Y	Y	Y	N	Y	Y	Y	Y	Y	Y	Y	Y	Y	14
Boye *et al.*, 2014^26^	Y	Y	N	Y	N	N	Y	N	Y	N	Y	Y	Y	Y	9
DeLuca *et al.,* 2020^28^	Y	Y	N	Y	N	Y	Y	N	Y	N	Y	N	Y	Y	9
Harvey *et al.,* 2017^29^	Y	Y	N	Y	N	N	Y	N	Y	N	Y	Y	Y	Y	9
Prabhakaran *et al.,* 2020^30^	Y	Y	N	Y	N	N	Y	N	Y	N	Y	N	Y	Y	8
Teo *et al.,* 2018^32^	Y	Y	N	Y	N	Y	Y	Y	Y	N	Y	Y	Y	Y	11
Tracy *et al.,* 2020^33^	Y	Y	Y	Y	N	Y	Y	Y	Y	N	Y	Y	Y	Y	12

*Note.* N= no; Y= yes. Quality Assessment Rating:^53^^,^^54^ For reviews (*) STRONG, Total score 8-10; MODERATE, Total score 5-7; WEAK, Total Score <4. For studies (**) STRONG, Total score 11-14, MODERATE, Total score 8-10, WEAK, Total Score < 7. The following items were considered for the literature reviews: 1) Research question, 2) Inclusion criteria, 3) Comprehensive search strategy, 4) Adequate number of years, 5) Level of evidence of studies, 6) Quality assessment of studies, 7) Are results transparent? 8) Appropriateness of combining study results (e.g., test of homogeneity), 9) Weighting, 10) Interpretation of results. In terms of each study, the following items were evaluated: 1) Objective clearly stated, 2) Study population, 3) Participation rate at least 50%, 4) Inclusion/exclusion criteria, 5) Sample size justification, 6) Exposure measured prior to outcome measurement, 7) Timeframe, 8) Categories/degree of exposure considered, 9) Independent variables clearly defined, valid, reliable 10) Exposure(s) assessed more than once, 11) Outcome measures clearly defined, valid, reliable, 12) Outcome assessors blinded to exposure, 13) Loss to follow-up <20%, 14) Key potential confounders measured and adjusted for.

### Life-Course Determinants of Frailty

Abdulle and colleagues found that when comparing frail with non-frail individuals, there were significant differences in several factors related to mild TBI.^25^ Overall, frail individuals were older, had worse injury severity scores, worse pre-mental health, more comorbidities, higher scores in anxiety and depression, worse physical, mobility, and psychosocial impairment scores, lower satisfaction with life scores, and lived alone.^25^ These results are concordant with those reported by Tracy et al., who found significant differences based on specific factors related to frailty in TBI.^33^ Among TBI patients, 61.6% (*n* = 1450) were not frail, 19.3% (*n* = 454) were mildly frail, and 19.1% (*n* = 448) were moderate to severely frail. Higher frailty scores were associated with increasing age, increasing Glasgow Coma Scale (GCS) scores, and decreasing Injury Severity Scores (ISS).^33^ Additionally, Brown *et al. found that* cognitive frailty index correlates with age.^27^

### Domains of Frailty

*Physical Domain of Frailty.* Age-related changes influence immune function and induce central nervous system changes/inflammatory response. ^31^ Frailty in older adults is associated with Vitamin D deficiency.^34^^,^^35^ Further, progesterone has been shown to modulate aquaporins/reduce edema and reduce glutamate toxicity, helping to reduce cell damage/decreased function among people with TBI.^36^ In addition, Tracy *et al.* found an association between moderate to severe frailty and acute kidney injury (AKI) (OR)= 2.06 [95% CI 1.07-3.99, p= .03] and concluded that frailty is predictive of AKI in people with TBI.^33^

*Social Domain of Frailty.* Harvey *et al.* found that community-dwelling TBI patients >65 years old experienced better outcomes than residential aged care facility (RACF) residents.^29^ These outcomes included an increased likelihood of experiencing falls related to furniture (21.4% vs. 9.9%), hemorrhage (82.5% vs. 73.7%), and death (23.1% vs. 14.9%) for RACF compared to community-dwelling, respectively. In addition, Boye *et al.* determined that in a sample of 5,880 individuals who visited the emergency room due to a fall, falls were likely to have occurred indoors compared to outdoor environments, and the prevalence of sustaining TBI post-fall was 4%.^26^

#### Psychological Domain of Frailty

*Cognition.* Mild cognitive impairment or dementia is associated with fall-related TBI admission (3.31 [95% CI 1.68-6.51], *p*<0.001).^32^

*Mood.* Abdulle *et al.* discovered that emotional distress was present in 50% of frail patients, compared to 20% of non-frail patients, in a sample of patients >60 years old at 3 Level 1 trauma centers.^25^

*Coping.* Coping influences an individual's ability to recover from a TBI. For example, positive psychology can enhance the healing process among people with TBI.^37^

### Adverse Health Outcomes

Frail older adults are more likely to experience poorer long-term outcomes from TBI.^25^ Abdulle and colleagues identified the effect of frailty and early postinjury measures on the long-term outcome after mild TBI in older patients (>60 years).^25^ The researchers learned that frailty (OR = 2.1 [95% CI: 1.59-2.77]) and early complaints (OR = 1.13 [95% CI: 1.01 - 1.27]) were stronger predictors of unfavorable functional outcomes. Together these predictors explained the 46% variance in the unfavorable functional outcomes measured with the Extended Glasgow Outcome Scale (GOSE). Age, early anxiety, and depression were not significant predictors of the long-term outcome within this cohort.^28^ Additionally, in recovery post-injury, the majority (72%) of non-frail older patients recovered completely from posttraumatic events compared to 24% of frail older patients.^25^

Tracy and colleagues reported that rates of falls as the primary traumatic mechanism increased as frailty scores increased.^33^ Moreover, patients with greater frailty had lower rates of immediate triage to the operating room following initial presentation to the emergency department.^33^ Prabhakaran et al. found that in a sample of venous thromboembolism (VTE) patients who were >65 years old, both frailty, measured by the modified frailty index (*p* < .001) and severe TBI (*p* <.001) were independent predictors of VTE development in the elderly.^30^ Frailty was the strongest independent risk factor for developing venous thromboembolism after trauma among older adults (OR=2.0, 95 % CI:1.82-2.28]).^30^ Furthermore, the study showed that TBI in older adults increased the time to initiate VTE prophylaxis, creating a risk of pulmonary embolism in the study population, in contrast to the early start of VTE prophylaxis in lower extremity injuries.^30^

Brown *et al.* found that poor health years prior to the injury and depression/fatigue shortly after injury are associated with poorer outcomes. Additionally, fall-related TBI is associated with a decline in ADLs, polypharmacy, and specialist outpatient clinic appointments (*p*<0.001).^32^

## Discussion

The results of this review indicate that outcomes resulting from TBI are closely linked to the physical, psychological, and social domains of frailty. Using this framework to understand frailty in the context of TBI, especially in older populations, can help clinicians anticipate patient outcomes and improve care plans. Although the quality of the majority of studies was good and received either a moderate or strong rating, there is a low volume of research conducted on this topic, indicating more studies are needed. Additionally, some of the included studies are quite narrow in focus, making it difficult to generalize findings.

According to this literature review, various life course determinants of frailty may influence the occurrence of a TBI (e.g., lifestyle and living environment may increase an individual's susceptibility to sustaining a TBI).^29^ Conversely, certain life course determinants may also influence the prognosis of TBI patients (e.g., age and sex). For instance, in frail older adults, old age may impair an individual's ability to recover quickly from TBI and lead to lingering symptoms and functional decline.^25^ Thus, TBI is both a risk factor for and a result of frailty. 

Examining the symptoms seen of TBI in frail populations can help to determine expected functional outcomes. Additionally, it must be acknowledged that the inconsistent use of measurement tools to determine frailty. A systematic and uniform tool to assess frailty would add more reliability to the research. The neurochemical imbalances observed in TBI (e.g., blood-brain barrier disruption, mood alterations) and frailty (e.g., increased susceptibility to falls, vitamin D deficiency, AKI) have implications for long-term health and rehabilitation outcomes. For example, an increased negative mood has been correlated with poorer health outcomes and the ability to recover from TBI.^38^ Frailty increases an individual's likelihood of experiencing depressive symptoms, which has been linked to a reduction in the quantity of monoamines neurotransmitters (e.g., dopamine, noradrenaline, serotonin) in synaptic clefts and depressive pathophysiology.^39^^,^^40^ Sustained expression of these neurochemical imbalances can enforce and prolong depressive symptoms, leading to increased frailty and poor health outcomes.

TBI results in notable biological and neurochemical alterations. The integrity of the blood-brain barrier (BBB) becomes compromised, resulting in a shift from the normally highly regulated transcellular transport to paracellular transport. Thus, more solutes can readily enter the brain and affect functioning.^41^ The aforementioned has implications related to cognition, mood, and motor function. Various biomarkers such as miRNAs (e.g., miR-124-3p) tau, and UCH-L1 that are implicated in the neuroinflammatory process of TBI, have been linked to neurodegeneration.^42^^-^^45^ The expression of S-100ß, a beta-tau protein, promotes oxidative stress and further exacerbation of injury.^46^ S-100ß is also linked with the formation of Aß plaques, which are histologically characteristic of Alzheimer's Disease.^47^ Neurodegenerative disease (e.g., Alzheimer's, dementia) has been linked to risk for fall related TBI,^32^ and may impair the healing process and ability to rehabilitate from injury.^48^ Neurodegenerative disease is also a hallmark characteristic of frailty.^49^

Our results bring to the forefront numerous considerations in frail TBI patients. In the physical domain, the impact of vitamin D deficiency and kidney injury were highlighted as being problematic, and also contribute to the damaging physiological cascade detailed above. Socially, skilled nursing facility placement was also shown to contribute to patient outcomes. Dijkers and colleagues found that older adults had lower motor functioning scores upon rehab discharge despite similar functioning scores upon admission.^48^The previous suggests that frailty may play a role in the impaired healing process and functional improvement. 

Psychologically, a patient’s cognitive function, ability to cope with the injury (e.g., positive psychology), and mood were all shown to impact findings; however the literature is limited. Other studies, such as Dijkers et al., have found that older adults have low cognitive functioning ability scores upon rehab discharge after a TBI.^48^ Mosenthal *et al.* found that functional ability is lower in older adults sustaining mild TBI when compared to adults ages 18-64.^50^ Decreased cognitive ability (e.g., mild cognitive impairment or dementia) may also increase the risk for fall-related TBI.^32^ From a clinical standpoint, this highlights the importance of monitoring for and addressing each of the domains of frailty and initiating appropriate interventions. For example, extra attention may be given to discharge instructions, teaching, and follow-up to a patient who is more frail. Both cognitive stimulation and monitoring for adverse psychological effects may also be appropriate interventions. From a research standpoint, more rigorous studies in frail TBI patients would enhance the literature (e.g., intervention studies focusing on symptoms or physical/cognitive outcomes).

Mood should be also considered as relevant for psychological frailty among older adults who have experienced a TBI, as emotional distress can be present among frail individuals. Consistent the results reported in this literature review, Kristman and colleagues found that patients reporting depression and fatigue shortly after sustaining TBI went on to experience poorer outcomes.^38^ Poorer health (e.g., pre-existing psychological symptoms of frailty) may influence the nature of the brain injury, and after TBI, the psychological compromise may be accelerated.^40^ Effective interventions for frail TBI patients may be able to enhance the healing process through promoting positive psychology and assist patients with coping with the sequelae of the injury.

The findings of this review in terms of adverse outcomes are consistent with previous literature on TBI. For example, according to Rothweiler et al, increased dependency, change in living circumstances, and mortality 1 to 2 years after TBI were all linked to increasing age at the time of injury.^51^ Testa et al. also concluded that older patients and those with TBI are likely to become physically and financially dependent on others.^52^ However, our results also suggest that frailty may be an even stronger predictor of TBI outcomes than age.^28^ This antecedent is important for further exploration as it has implications for patient management and understanding of TBI outcomes.

Limitations. This review seeks to understand the multifaceted, multidimensional nature of frailty in the context of TBI in older adults. Our results indicate that physical, social, and psychological considerations are relevant when considering clinical outcomes for TBI patients that are also experiencing the progression of frailty. However, our review also contains limitations. There are potential resources not included in this review that could have contributed to a better understanding of the relationship between TBI and frailty. For instance, gray literature and different language publications could have been included. In addition, the search terms chosen and databases used may have limited the scope of information identified. Furthermore, a small number of final articles was yielded, which may be in part because both TBI and frailty had to be concurrently examined for the review to warrant inclusion in our final results, and there is a shortage of literature on this topic. Thus, more research examining frailty and TBI is needed, especially considering that frailty and TBI may be co-occurring or increase an individual's susceptibility to experiencing the other condition.

## Conclusion

TBI and frailty are significant health concerns in older adults that are likely to persist as the population ages. This literature review provides some insights to support the relationship between TBI and frailty and to improve nursing practice and knowledge. Using a multidimensional model to understand TBI that encompasses physical, social, and psychological domains of frailty has the potential to improve outcomes for TBI patients. Further research is needed to test and understand this model in various populations. Appropriate interventions should be developed to address frail older adults' physical, social, and psychological needs that sustain TBI. Nurses and clinicians caring for older adults can use the multidimensional model of frailty to provide patient care to TBI patients to improve clinical outcomes and recovery from TBI.
